# Supplementary figures and images for: Intraspecies Transcriptional Profiling Reveals Key Regulators of Candida albicans Pathogenic Traits
Source: mBio. 2021 Apr 20;12(2):e00586-21. doi: 10.1128/mBio.00586-21 (PMC8092256; doi:10.1128/mBio.00586-21)

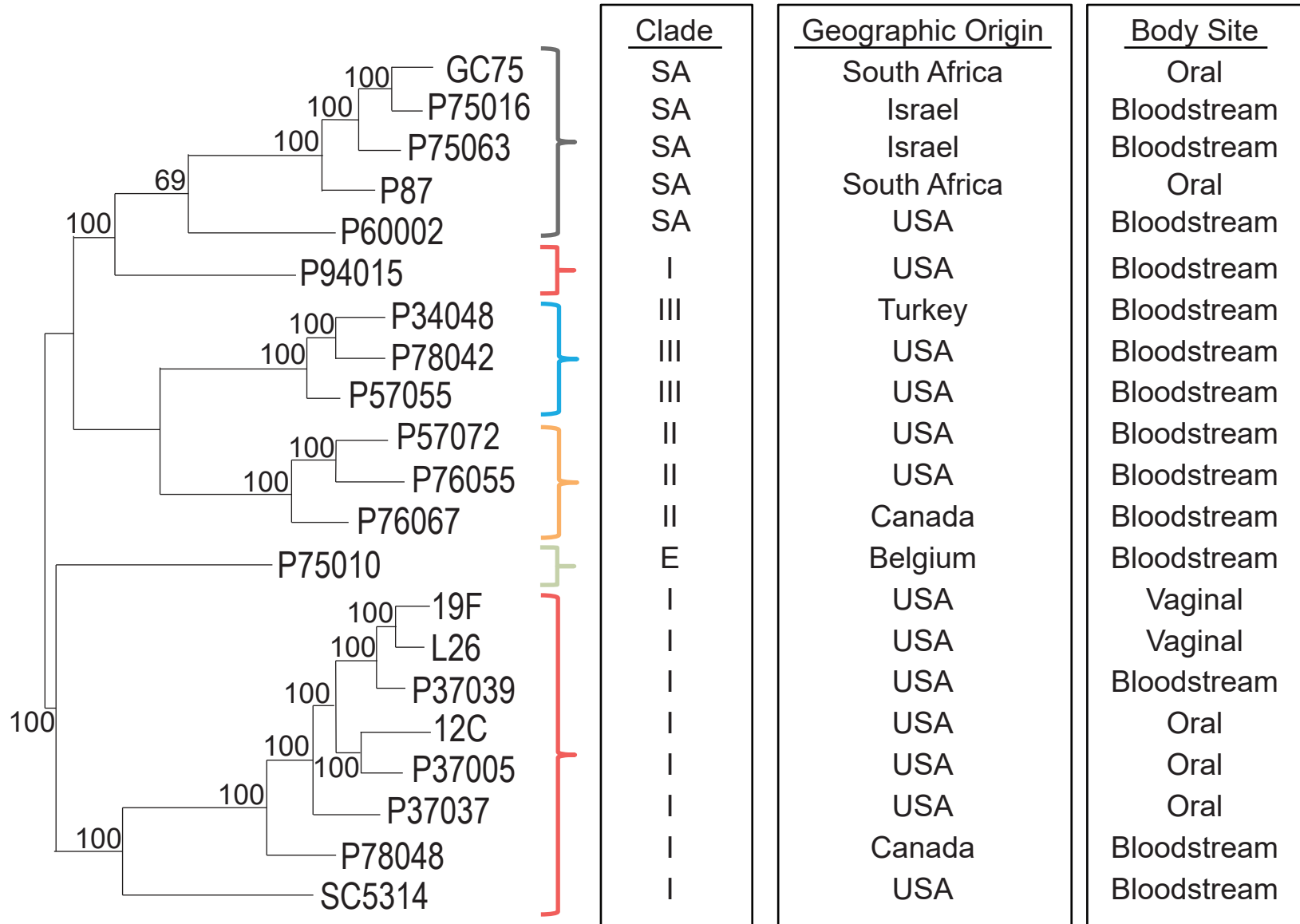

Supplement: FIG S1 [file mBio.00586-21-sf001.pdf]

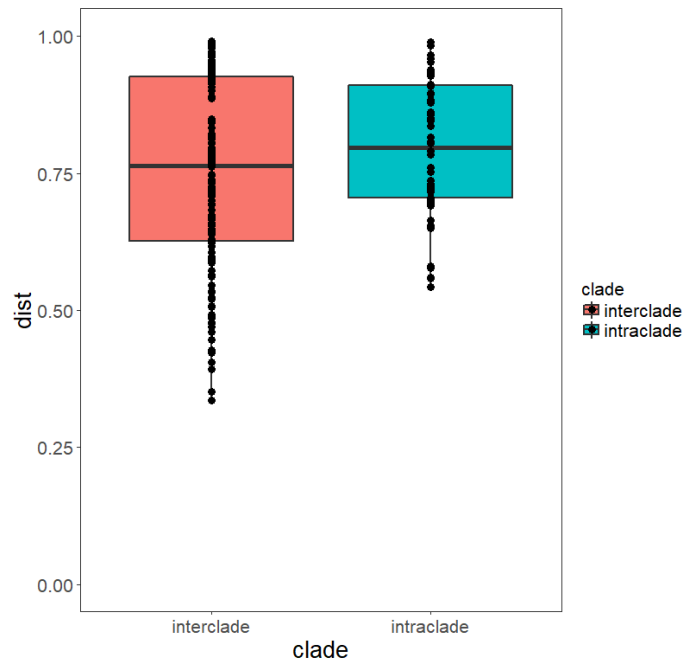

Supplement: FIG S3 [file mBio.00586-21-sf003.pdf]

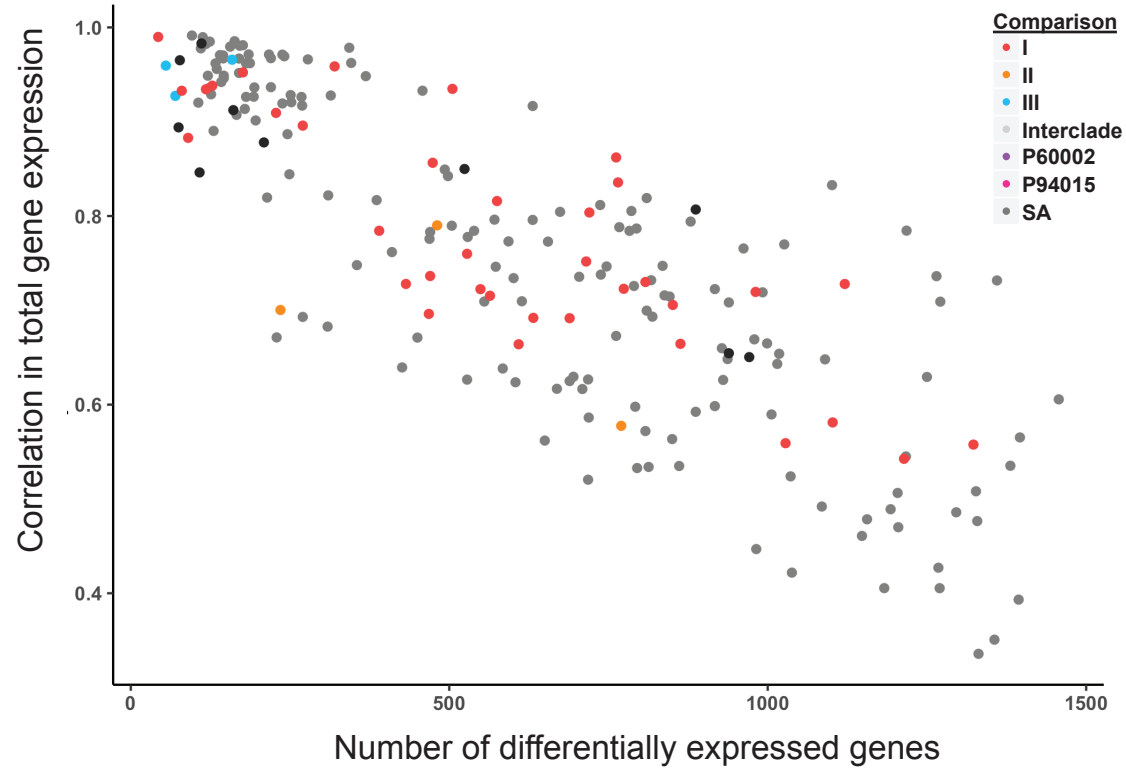

Supplement: FIG S4 [file mBio.00586-21-sf004.pdf]

A

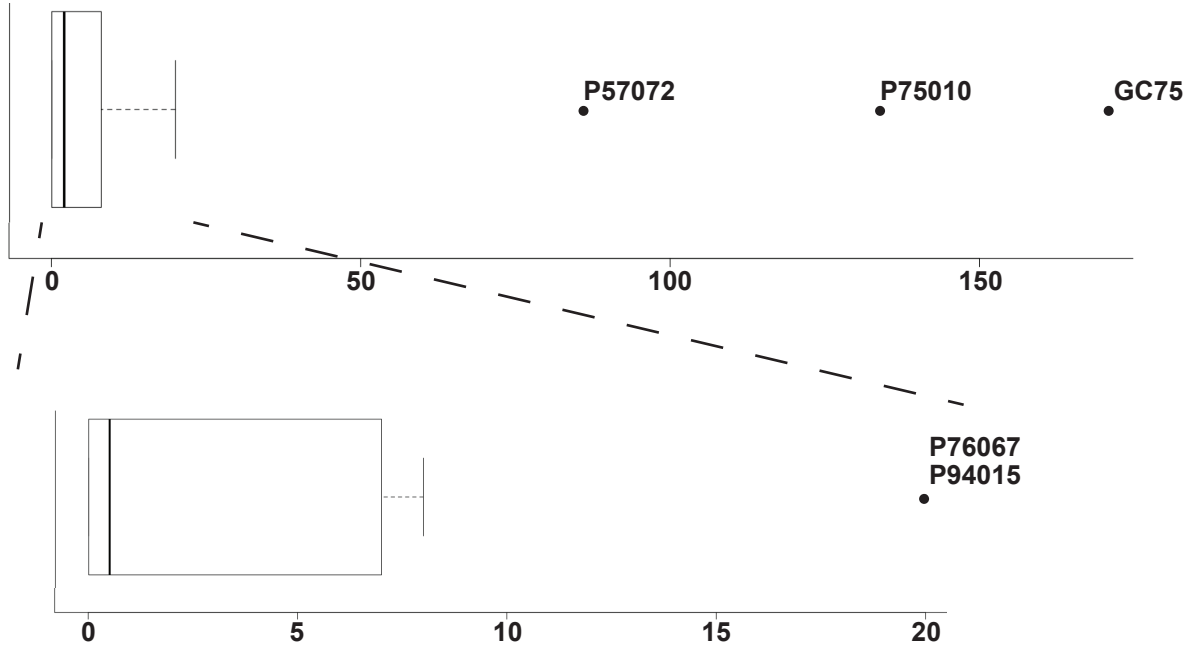

B

| Isolate | Strain-Specific Genes |
|---------|-----------------------|
| 12C     | 0                     |
| 19F     | 0                     |
| GC75    | 171                   |
| L26     | 0                     |
| P34048  | 3                     |
| P37005  | 0                     |
| P37037  | 0                     |
| P37039  | 0                     |
| P57055  | 0                     |
| P57072  | 86                    |
| P60002  | 7                     |
| P75010  | 134                   |
| P75016  | 2                     |
| P75063  | 1                     |
| P76055  | 8                     |
| P76067  | 20                    |
| P78042  | 8                     |
| P78048  | 0                     |
| P87     | 7                     |
| P94015  | 20                    |
| SC5314  | 0                     |

Supplement: FIG S5 [file mBio.00586-21-sf005.pdf]

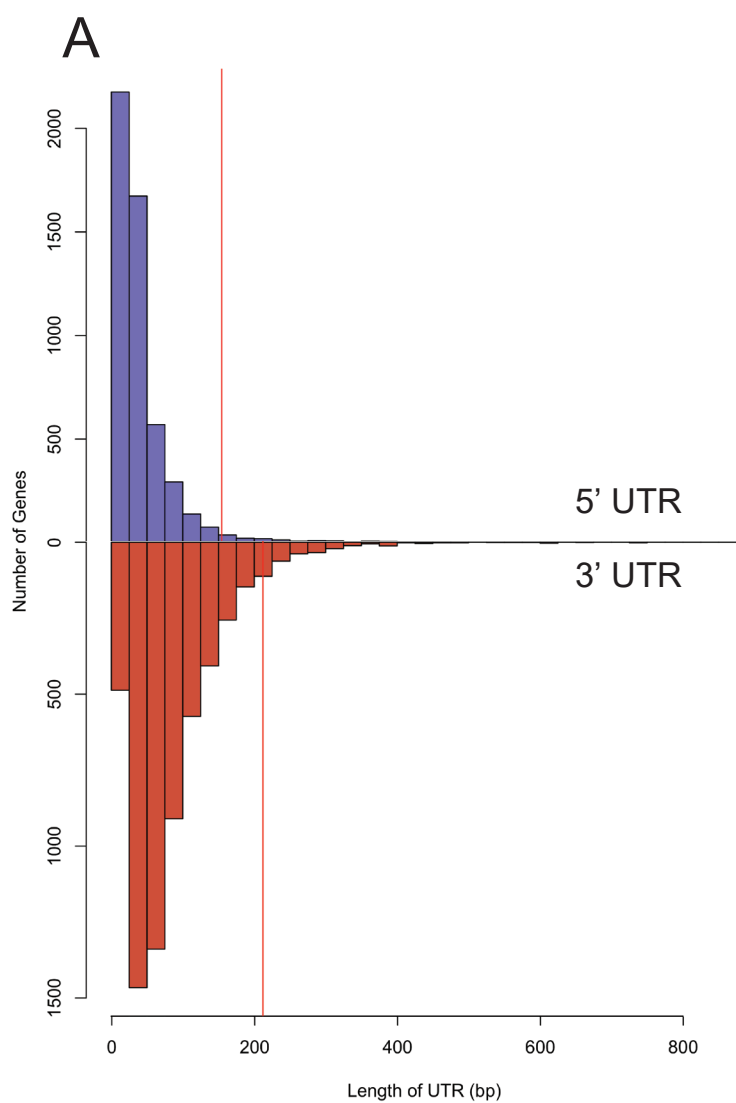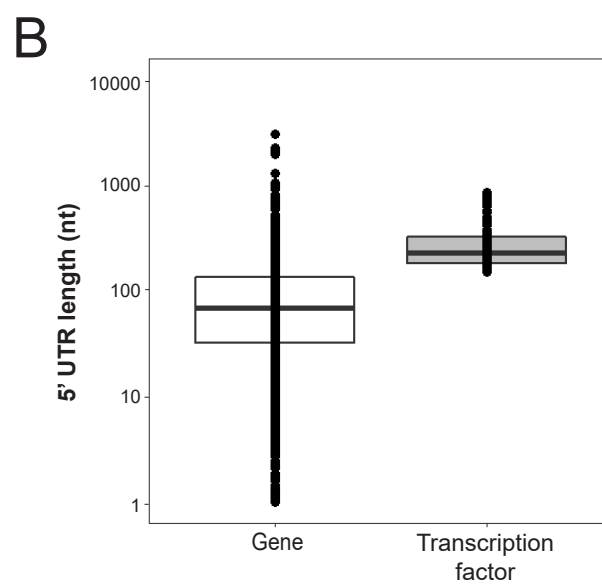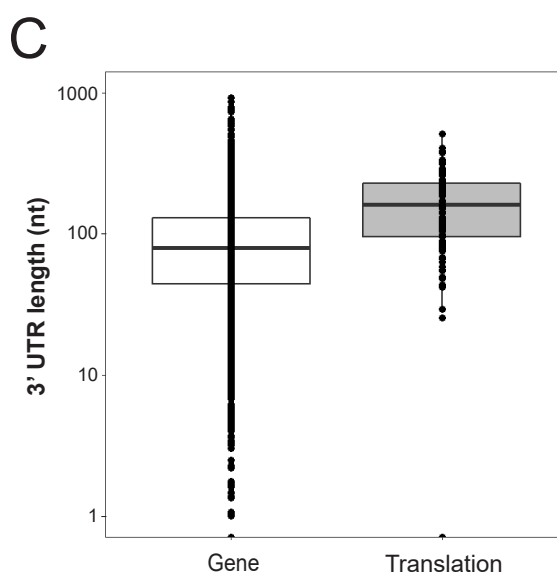

Supplement: FIG S6 [file mBio.00586-21-sf006.pdf]

A

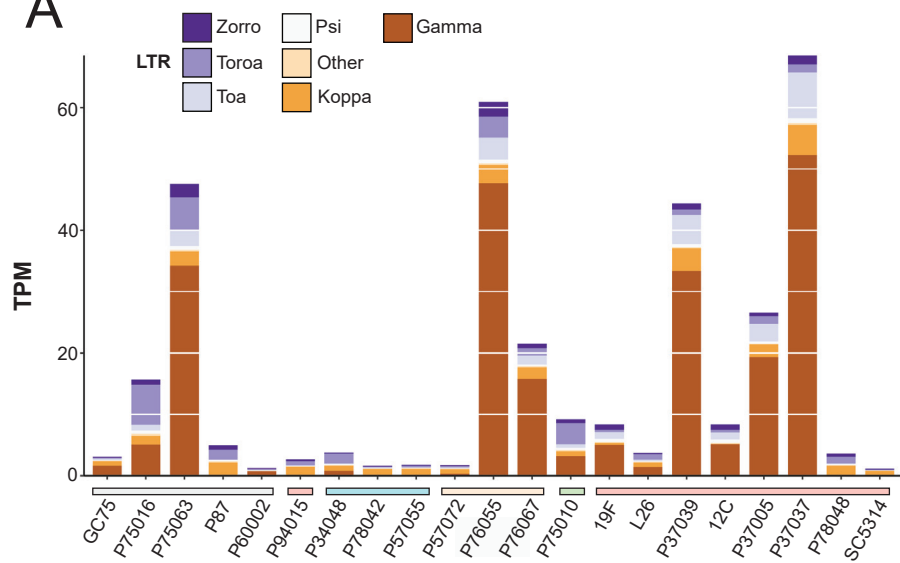

B

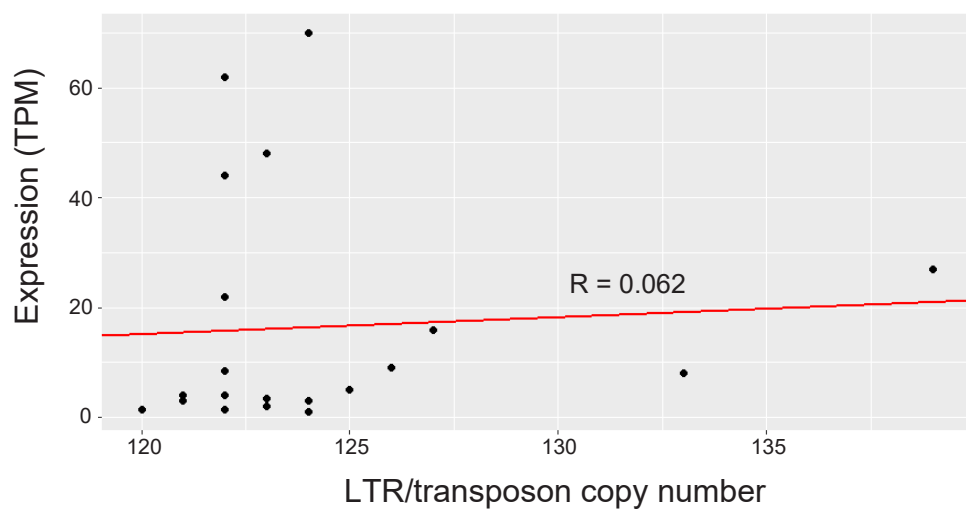

Supplement: FIG S7 [file mBio.00586-21-sf007.pdf]

**A**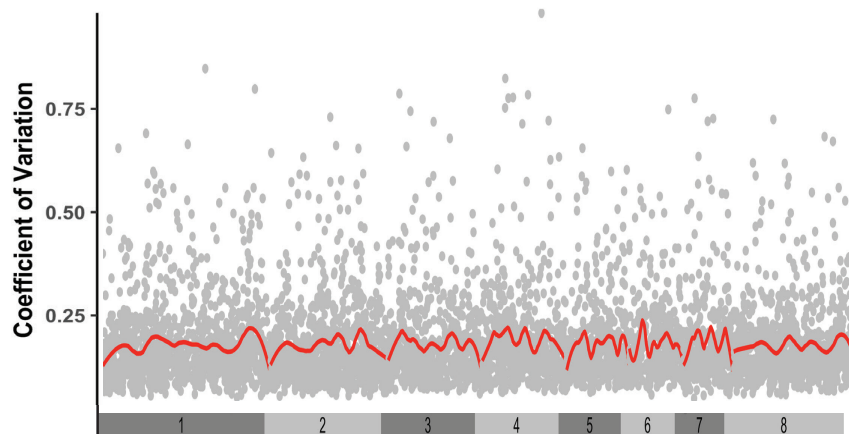**B**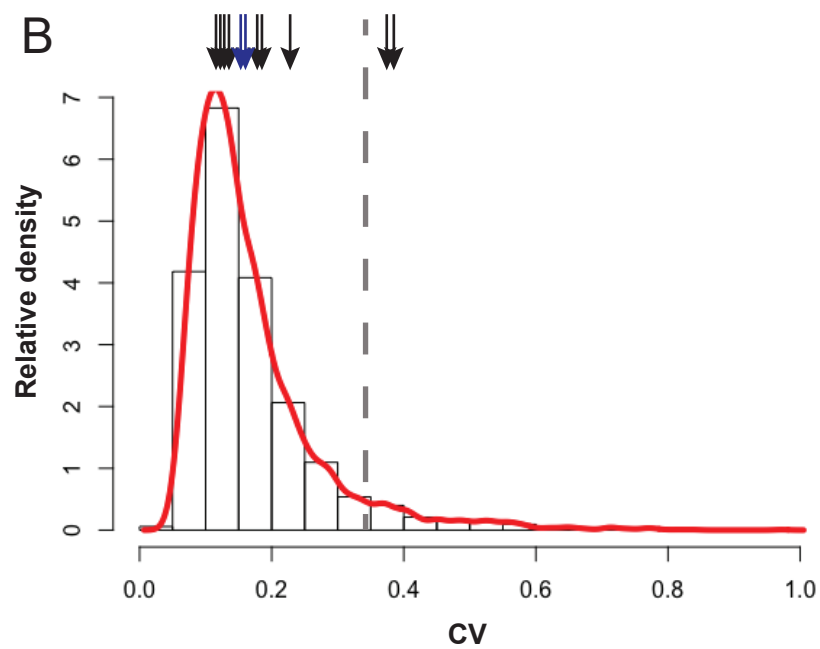

Supplement: FIG S8 [file mBio.00586-21-sf008.pdf]

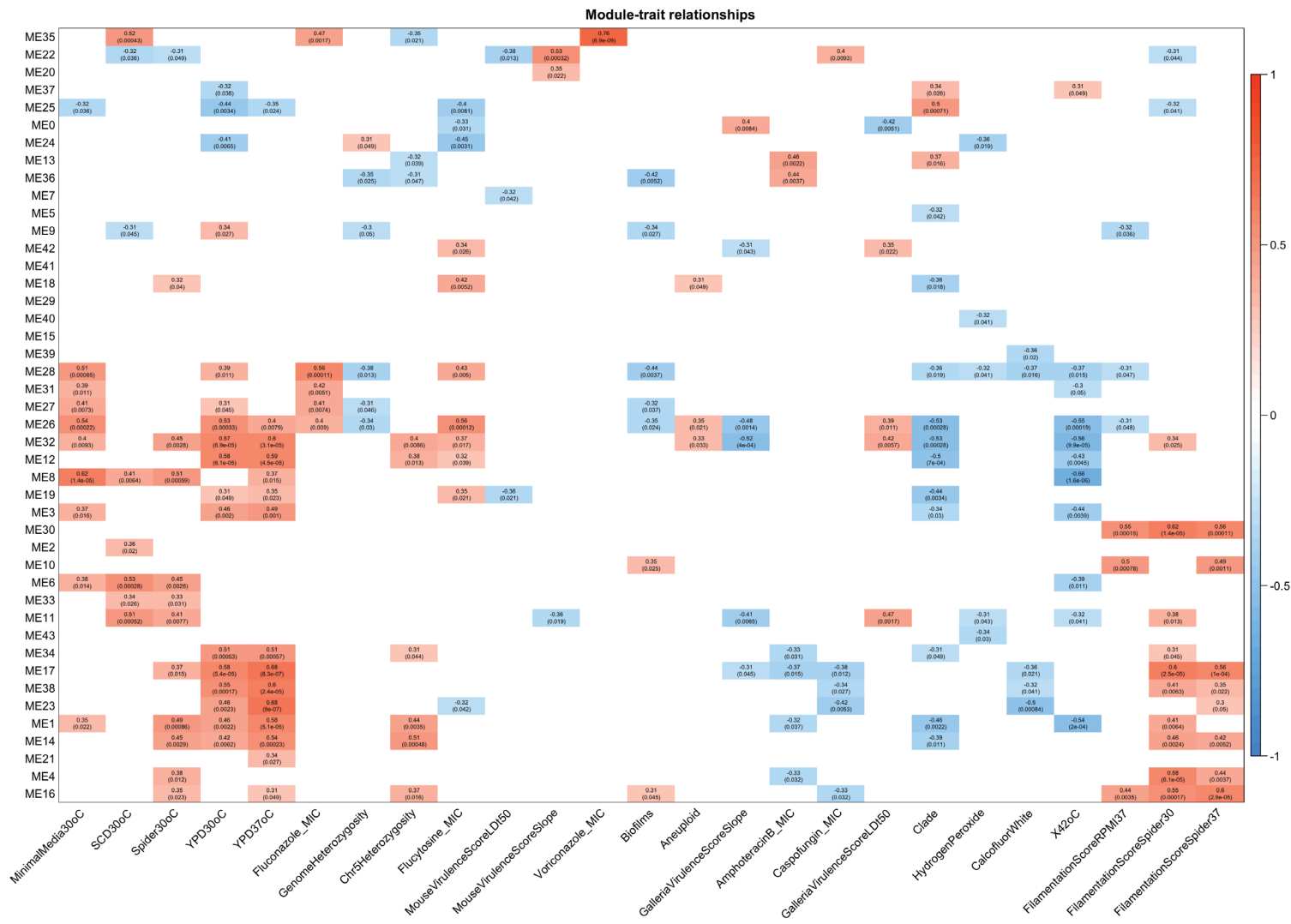

Supplement: FIG S9 [file mBio.00586-21-sf009.pdf]

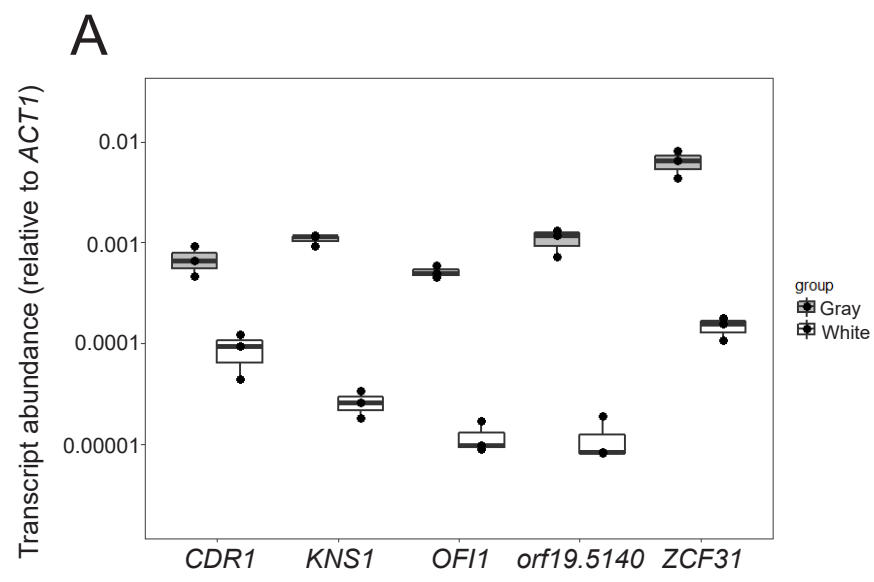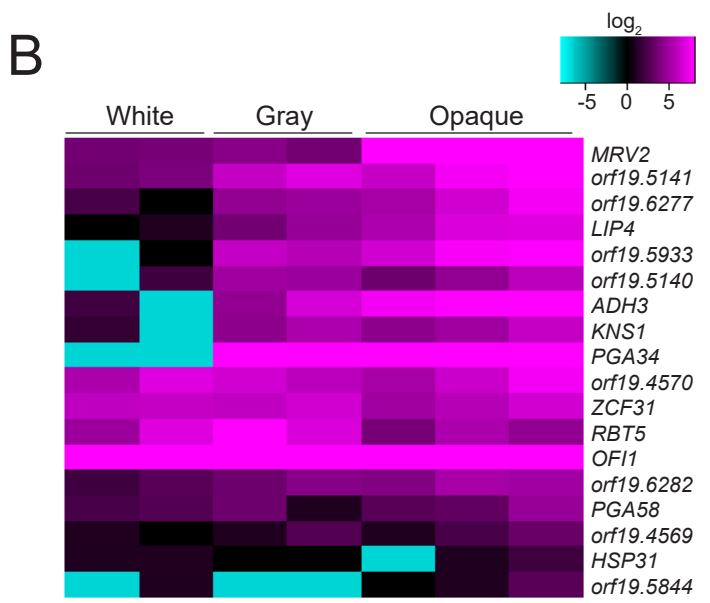

Supplement: FIG S10 [file mBio.00586-21-sf010.pdf]
