# Supplementary material for: Intraspecies Transcriptional Profiling Reveals Key Regulators of Candida albicans Pathogenic Traits
Source: mBio. 2021 Apr 20;12(2):e00586-21. doi: 10.1128/mBio.00586-21 (PMC8092256; doi:10.1128/mBio.00586-21)
Supplement: FIG S2 [file mBio.00586-21-sf002.pdf]

A

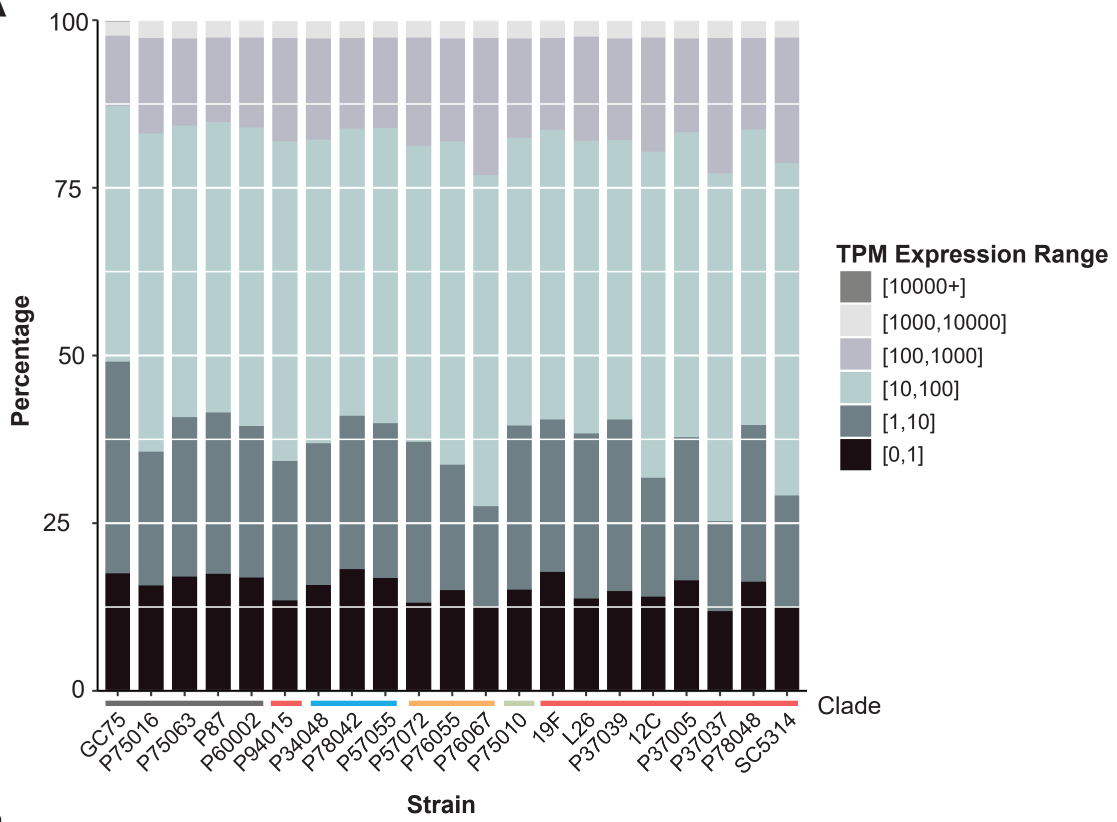

B

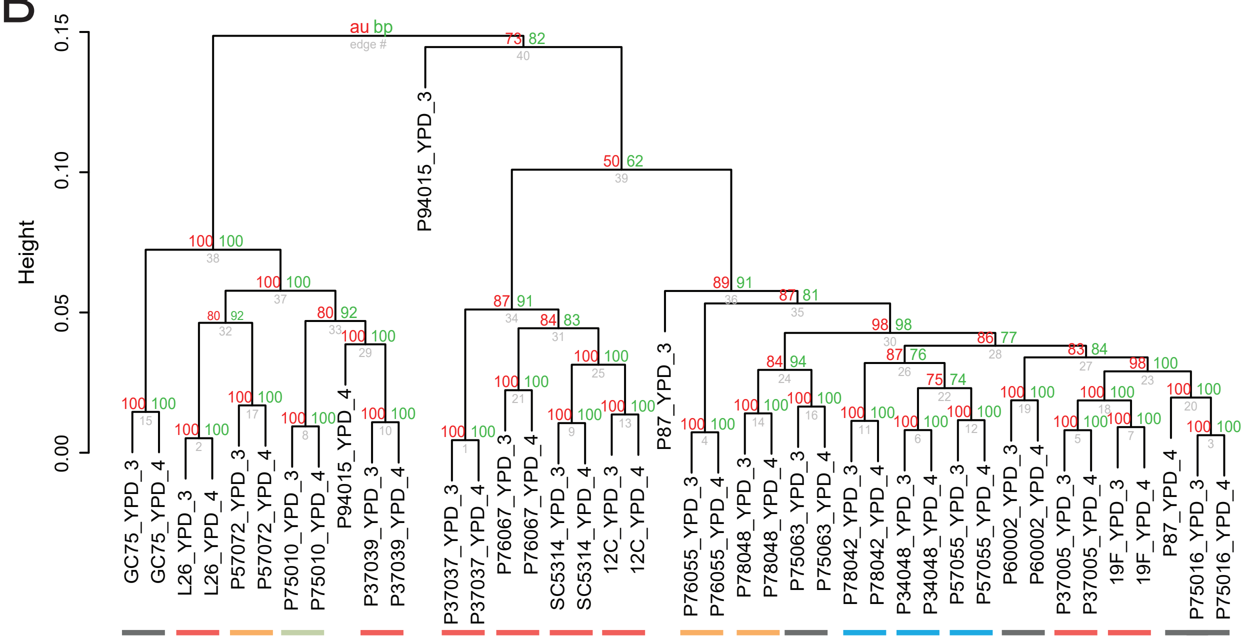

C

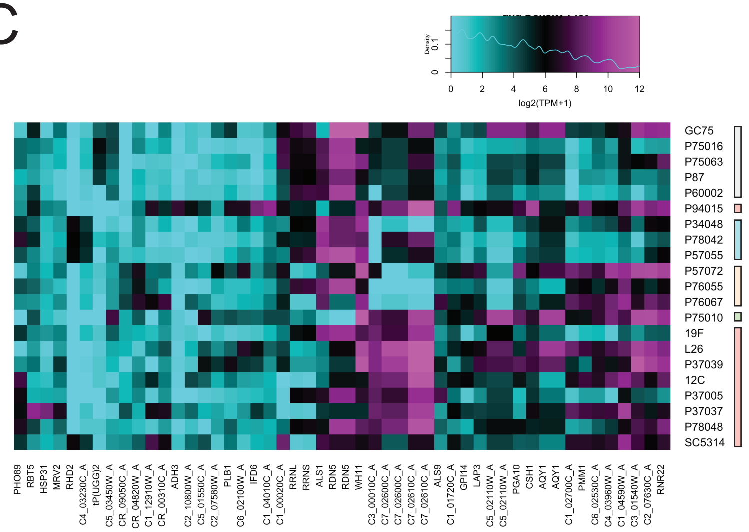

D

Process	Genes included (of 31)	Total possible	Probability (q)
regulation of transcription from RNA polymerase II promoter by glucose	3	12	5.03E-3
regulation of transcription by glucose	3	14	8.28E-3

Significantly correlated genes:

C6_03800C	C4_07040W	C1_13690C
**VPS22**	TOM22	FGR14
HAL21	PEP1	C5_00130C
MNT1	RPL30	GEM1
OPT4	AGP3	CR_07300W
**STB3**	ZCF19	C3_05900W
LEU4	C5_01410C	C1_04940C
C5_03710C	CR_03780C	C6_02310W
C2_00890W	C1_02000W	C6_00890W
**TPK2**	MNN47	
SNZ1	TVP18	
